# Exploring alternative routes for oxygen administration

**DOI:** 10.1186/s40635-016-0108-z

**Published:** 2016-10-10

**Authors:** Elisa Damiani, Alex Dyson, Lucia Zacchetti, Abele Donati, Mervyn Singer

**Affiliations:** 1 Anesthesia and Intensive Care Unit, Department of Biomedical Sciences and Public Health, Università Politecnica delle Marche, via Tronto 10/A, 60126 Torrette di Ancona, Italy; 2 Bloomsbury Institute of Intensive Care Medicine, Division of Medicine, University College London, London, UK; 3 Department of Anesthesiology and Intensive Care, Fondazione IRCCS, Cà-Granda Ospedale Maggiore Policlinico, Milan, Italy

**Keywords:** Oxygen therapy, Hypoxemia, Tissue oxygenation

## Abstract

**Background:**

Hypoxemia may compromise cell metabolism and organ function. Supplemental oxygen (O_2_) at high concentrations may prove ineffective, and issues relating to hyperoxia, barotrauma, mechanical ventilation, and extracorporeal oxygenation are well documented. Old reports suggest the potential safety and efficacy of alternative routes for O_2_ administration, such as intravenous or intestinal. We re-explored these routes in rat models of hypoxemia.

**Methods:**

Hypoxemia was induced in spontaneously breathing, anesthetized rats by breathing a hypoxic gas mix (FiO_2_ 0.1). Pilot studies infusing pure O_2_ gas caused early death, likely due to pulmonary embolism. Instead, rats (*n* = 6/group) were given intravenous O_2_ via a continuous infusion of pre-oxygenated Hartmann’s solution (10 ml/kg/h) for 3 h with normal Ringer’s lactate used in control animals. In separate experiments (*n* = 8/group), bowel intraluminal oxygenation was assessed with pure O_2_ administered through a cannula placed into the jejunal lumen at a dose of a 15 ml/kg bolus followed by a continuous infusion of 50 ml/kg/h; no treatment was given to controls. Echocardiography, arterial blood gas analysis, mean arterial pressure, muscle and liver tPO_2_, muscle microvascular perfused vessel density, and urine output were measured.

**Results:**

Administration of oxygenated Hartmann’s solution (PO_2_ of solution at end-experiment = 87.5 ± 1.7 kPa) was safe but did not increase either systemic or tissue oxygenation. Similarly, the administration of bowel O_2_ was safe but did not improve neither systemic nor liver oxygenation.

**Conclusions:**

In this rat model of hypoxemia, the intravenous infusion of gaseous O_2_ was unfeasible as it induced early mortality. Although safe, both intravenous infusion of oxygenated Hartmann’s solution and bowel O_2_ administration were unable to improve arterial or tissue oxygenation.

## Background

During hypoxemia, the low tissue oxygen (O_2_) supply may compromise cellular aerobic metabolism and determine organ dysfunction. Supplemental O_2_ at high concentration, with or without supported ventilation, is usually administered for hypoxemic respiratory failure. However, this conventional approach may prove inadequate when gas exchange through the alveolar-capillary membrane is severely impeded, e.g., severe acute respiratory distress syndrome [1]. In addition, adverse effects of excessive inspired O_2_ administration and mechanical ventilation are well documented, including hyperoxia- and ventilator-induced acute lung injury [2]. Extracorporeal membrane oxygenation is an alternative albeit invasive modality that is also associated with a significant complication rate including bleeding, thrombosis, and infection [3].

A century ago, Tunnicliffe and Stebbing reported rapid and safe symptomatic relief of cyanosis in patients following the administration of intravenous O_2_ gas through a peripheral venous cannula at a rate of 600–1200 ml/h [4]. The authors did however recommend that “irregularity of the pulse” and “dilatation of the right side of the heart” are indications to stop the gas administration [4]. In 1888, John Harvey Kellogg at the Battle Creek Sanitarium in Michigan showed that O_2_ may be rapidly absorbed through the intestinal mucosa and encouraged the use of O_2_ enemata as a remedy for respiratory failure-induced cyanosis and reported several successfully treated cases [5]. These routes for O_2_ administration warrant re-exploration as potentially easy and cheap alternatives to conventional O_2_ therapy. We therefore sought to determine the safety and efficacy of intravenous and intestinal O_2_ administration in a rat model of hypoxemia.

## Methods

All experiments were performed according to Home Office (UK) guidelines under the 1986 Scientific Procedures Act with local (University College London) ethics committee approval (PPL 70/8290). Spontaneously breathing male Wistar rats (300–350 g body weight) were anesthetized by 5 % isoflurane in room air in an induction chamber and then placed on a heated mat to maintain rectal temperature at 37 °C throughout. Under 2 % isoflurane anesthesia, the left common carotid artery and right internal jugular vein were cannulated. The arterial line was connected to a pressure transducer (Powerlab, AD Instruments, Chalgrove, UK) for continuous monitoring of mean arterial pressure (MAP). A tracheostomy was performed and connected to a T-piece to maintain anesthesia and vary the fraction of inspired O_2_ (FiO_2_). The bladder was exposed through a small laparotomy and cannulated using a 1.57-mm-OD tubing for drainage and quantification of urine output. A small incision was made on the external side of the right hind limb, and an 18-gauge guidance cannula was used to insert a tissue O_2_ tension (tPO_2_) sensor (Oxylite, Oxford Optronix, Milton Park, Oxon, UK) into the right vastus lateralis muscle [6]. For measurement of liver tPO_2_, the probe was placed into the airtight space between two of the liver lobes through a small midline laparotomy [6]. To enable microcirculatory assessment, a 2-cm skin incision was performed on the medial side of the left hind limb, and the perimysium was carefully separated from the muscle by blunt dissection in order to minimize tissue damage and bleeding [7].

In 16 animals (bowel O_2_ model), a 2-cm midline laparotomy was additionally performed to allow insertion of an 18-gauge cannula into the jejunum to which it was secured by a suture. The cannula was exteriorized to enable injection of O_2_, and the abdomen was closed with sutures. After instrumentation, isoflurane was reduced to 1.2 % for the remainder of the experiment. Euvolemia was obtained by intravenous administration of 4 ml/kg Hartmann’s solution followed by a continuous infusion of 10 ml/kg/h. The animals were allowed to stabilize for 60 min before baseline measurements were taken. The FiO_2_ was then reduced to 0.1 and maintained until the end of the experiment to induce hypoxemia. At the end of the study, the animals were sacrificed without regaining consciousness.

### Model 1: intravenous O_2_ administration

Pilot studies were performed to construct a model of intravenous administration of gaseous O_2_. The infusion of pure O_2_ gas at a slow rate of 2 ml/kg/h (approximately 0.67 ml/h) resulted in death within minutes. The demise was preceded by a sharp fall in MAP and dilatation of the right ventricle evidenced by echocardiography, suggesting acute heart failure due to pulmonary embolism. Therefore, this technique was abandoned.

As an alternative, oxygenated Hartmann’s solution was prepared by bubbling pure O_2_ gas into a bag of Hartmann’s solution until the fluid was fully saturated. An O_2_ tension (PO_2_) of 100 kPa within the solution was confirmed by gas analysis (ABL625 blood gas analyzer, Radiometer, Copenhagen, Denmark). The PO_2_ of the solution was re-assessed at end-experiment. After 60 min of hypoxemia, animals (*n* = 6/group) were randomized to receive a continuous infusion of 10 ml/kg/h of oxygenated Hartmann’s solution (IV-O_2_ group) or an equal volume of normal, non-oxygenated Hartmann’s solution (control group) over the following 3 h. Measurements were taken at baseline and at hourly intervals for 4 h.

### Model 2: bowel O_2_ administration

After 30 min of hypoxemia, eight animals (bowel O_2_ group) received an injection of 15 ml/kg of pure O_2_ gas into the jejunal lumen, followed by continuous administration at a rate of 50 ml/kg/h for 90 min. These volumes were determined in pilot studies as the maximum volumes not producing any signs of intestinal over-distension or ischemia. No oxygen treatment was administered to a control group (*n* = 8). Measurements were taken at baseline and at 30-min intervals for 120 min. Five animals per group were monitored for a further 2 h.

### Measurements

Echocardiography (Vivid 7, GE Healthcare, Bedford, UK) was performed to assess stroke volume (SV), heart rate (HR), and respiratory rate (RR). SV was calculated from the flow-velocity integral measured in the ascending aorta (assuming an aortic annulus radius of 1.4 mm [6]) by pulsed-wave Doppler. Arterial and venous blood samples (about 0.2 ml) were collected in heparinized capillary tubes for blood gas analyses (ABL625 analyzer), which included measurements of arterial base excess (ABE) and lactate. Cumulative urine output was measured at 120 min (bowel O_2_ model) and at the end of the experiment. Muscle and liver tPO_2_ were assessed using large-area-surface O_2_ sensors (0.7-mm diameter) connected to an Oxylite tissue monitoring system, as described previously [7]. The skeletal muscle microcirculation was evaluated with sidestream dark field videomicroscopy (Microscan, Microvision Medical, Amsterdam, NL), which enables real-time in vivo visualization of blood flow in microvascular beds [8]. Videos from five adjacent sites were recorded with adequate contrast, focus, and stability; an absence of pressure artifact was defined by preservation of venular perfusion. The perfused vessel density (PVD), representing the total length of vessels with a continuous/sluggish flow divided by the total surface of the image, was calculated offline using AVA 3.0 (Automated Vascular Analysis) software (MicroVision Medical) [9].

### Statistics

Statistical analysis was performed using Graph Pad version 5 (GraphPad Software, La Jolla, CA, USA). Normality of distribution was checked using the Kolmogorov-Smirnov test. Data are expressed as mean ± standard error or median [first to third quartile], as appropriate. Statistics on parametric data were performed using repeated measures two-way analysis of variance followed by Bonferroni’s post hoc test. Non-parametric data were analyzed using the Friedman test with Dunn’s post hoc test for multiple comparisons and the Mann-Whitney *U* test. A *p* value <0.05 was used to indicate statistical significance.

## Results

### Model 1: intravenous O_2_ administration

**Fig. 1:**
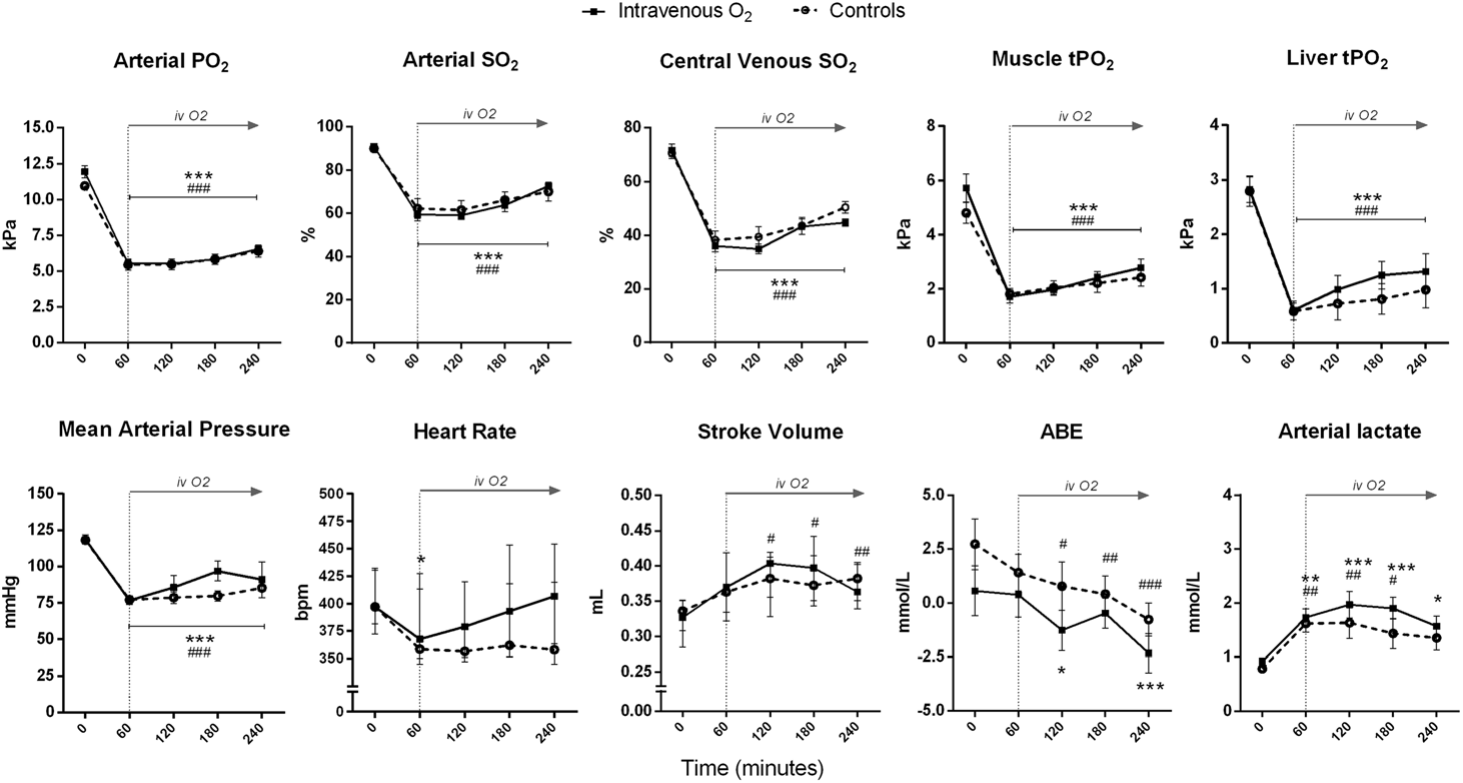
Effects of intravenous oxygenated Hartmann’s solution on oxygenation and hemodynamics in hypoxemic rats. Breathing 0.1 FiO_2_, rats (*n* = 6/group) received an intravenous infusion of 10 ml/kg/h of oxygenated (intravenous O_2_) or normal Hartmann’s solution (controls) over 3 h. Arterial O_2_ tension (PO_2_), arterial and central venous O_2_ saturation (SO_2_), muscle and liver tissue PO_2_, mean arterial pressure, arterial base excess (ABE), and lactate are expressed as mean ± standard error. Heart rate and stroke volume are expressed as median [first to third quartile]. *p < 0.05, **p < 0.01, and ***p < 0.001 versus baseline in the intravenous O_2_ group; ^#^ *p* < 0.05, ^##^ *p* < 0.01, and ^###^ *p* < 0.001 versus baseline in the control group (repeated measure two-way analysis of variance with Bonferroni’s post hoc test or Friedman test with Dunn’s post hoc test)

All animals survived until the end of the 4-h experiment. At baseline, no statistically significant differences were observed between the two groups in any of the variables measured. After 1 h of breathing 10 % O_2_, similar reductions in arterial PO_2_, arterial and venous O_2_ saturation (SO_2_), and tissue PO_2_ in the muscle and liver were observed in the two groups (Fig. 1). A similar decrease in MAP was observed in the two groups (*p* < 0.001 versus baseline). HR and SV tended to decrease and increase, respectively. Arterial lactate levels were significantly elevated at 60 min in both groups while ABE tended to decrease (Fig. 1). Respiratory rate tended to be elevated in both groups during hypoxia, leading to a significant reduction in arterial carbon dioxide tension (PCO_2_) and increase in pH (Table 1).

**Table 1:** Respiratory and microcirculatory data for model 1 (intravenous O_2_ administration)

	Baseline	60 min	120 min	180 min	240 min
Respiratory rate (*n*/min)					
IV-O_2_	65 [62–73]	71 [69–77]	75 [71–83]*	75 [71–81]*	72 [69–79]
Controls	77 [60–89]	83 [67–96]	81 [69–94]	85 [69–93]	84 [69–97]
PaCO_2_ (kPa)					
IV-O_2_	4.8 ± 0.1	3.8 ± 0.1**	3.5 ± 0.1**	3.5 ± 0.1**	3.3 ± 0.1**
Controls	4.8 ± 0.1	3.5 ± 0.1**	3.4 ± 0.2**	3.2 ± 0.1**	3.1 ± 0.1**
pH					
IV-O_2_	7.44 ± 0.01	7.52 ± 0.01**	7.53 ± 0.01**	7.53 ± 0.02**	7.53 ± 0.02**
Controls	7.47 ± 0.01	7.55 ± 0.01**	7.56 ± 0.01**	7.57 ± 0.01**	7.57 ± 0.01**
Perfused vessel density (mm/mm^2^)					
IV-O_2_	17.0 ± 0.7	19.5 ± 1.0***	18.5 ± 0.6	18.6 ± 0.4	18.5 ± 0.8
Controls	20.9 ± 0.7	24.6 ± 2.1	21.8 ± 1.0	20.8 ± 1.7	20.2 ± 0.9

*p < 0.01; **p < 0.001 versus baseline; ***p < 0.05 versus controls

The administration of oxygenated Hartmann’s solution (PO_2_ of solution at end-experiment = 87.5 ± 1.7 kPa) did not yield any significant improvement in blood or tissue oxygenation (Fig. 1). Respiratory alkalosis persisted until the end of the experiment in both groups (Table 1).

A non-significant increase in microcirculatory PVD was observed at 60 min in both groups (Table 1). No significant differences were found between the two groups throughout the experiment, apart from a lower value at 60 min in the IV-O_2_ groups as compared to controls (*p* = 0.011, Table 1).

Cumulative urine output at 240 min did not differ between the two groups (6.5 [5.5–18.9] ml/kg in the IV-O_2_ group and 5.8 [4.9–6.4] ml/kg in controls, *p* = 0.238).

### Model 2: bowel O_2_ administration

**Fig. 2:**
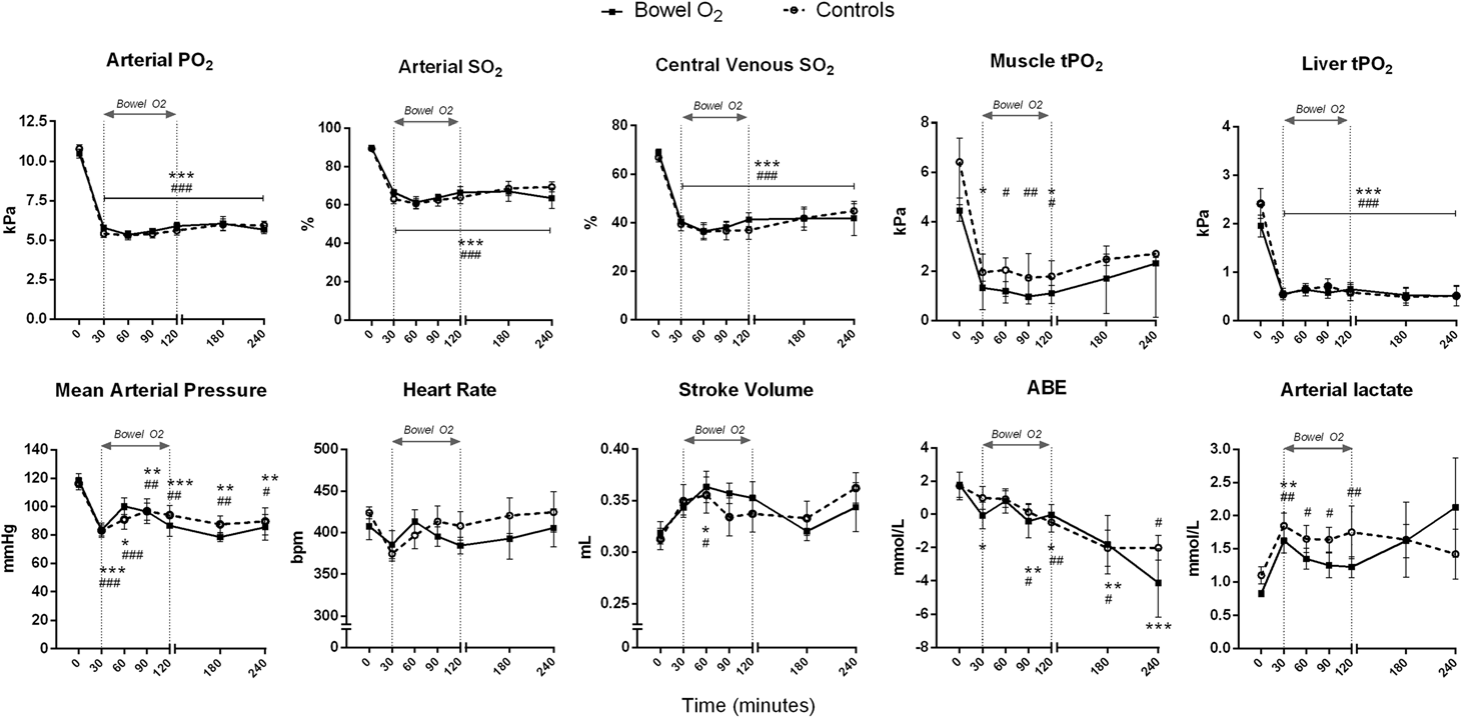
Effects of the administration of pure O_2_ gas into the bowel on oxygenation and hemodynamics in hypoxemic rats. Breathing 0.1 FiO_2_, rats were injected with pure O_2_ gas (15 ml/kg as a bolus followed by a continuous infusion of 50 ml/kg/h for 2 h) into the jejunum (bowel O_2_ group, *n* = 8). No oxygen treatment was given to a group of controls (*n* = 8). Five animals per group were monitored for a further 2 h after infusion discontinuation. Arterial O_2_ tension (PO_2_), arterial and central venous O_2_ saturation (SO_2_), liver tissue PO_2_, mean arterial pressure, heart rate, stroke volume, arterial base excess (ABE), and lactate are expressed as mean ± standard error. Muscle tPO_2_ is expressed as median [first to third quartile]. *p < 0.05, **p < 0.01, and ***p < 0.001 versus baseline in the bowel O_2_ group; ^#^ *p* < 0.05, ^##^ *p* < 0.01, and ^###^ *p* < 0.001 versus baseline in the control group (repeated measure two-way analysis of variance with Bonferroni’s post hoc test or Friedman test with Dunn’s post hoc test)

All animals survived until the end of the experiment. At baseline, no statistically significant differences were observed between the two groups in any of the variables measured. After 30 min of breathing 10 % O_2_, similar changes in arterial PO_2_, arterial and venous SO_2_, and tPO_2_ in the muscle and liver were observed in the two groups (Fig. 2). MAP was significantly reduced in both groups (*p* < 0.001 versus baseline); HR tended to decrease while SV tended to increase (Fig. 2). Arterial lactate levels were elevated in both groups (*p* < 0.01 versus baseline) while ABE was significantly reduced only in the bowel O_2_ group (*p* = 0.012 versus baseline) (Fig. 2). Respiratory rate increased following hypoxemia in both groups, leading to respiratory alkalosis (Table 2).

**Table 2:** Respiratory and microcirculatory data for model 2 (bowel O_2_ administration)

	Baseline	30 min	120 min	240 min
Respiratory rate (*n*/min)				
Bowel-O_2_	70 ± 3	81 ± 3*	81 ± 3*	82 ± 2*
Controls	69 ± 4	83 ± 4**	86 ± 5***	81 ± 7**
PaCO_2_ (kPa)				
Bowel-O_2_	4.7 ± 0.1	3.5 ± 0.1***	3.4 ± 0.1***	2.8 ± 0.2***
Controls	4.7 ± 0.2	3.5 ± 0.1***	3.2 ± 0.1***	2.8 ± 0.1***
pH				
Bowel-O_2_	7.47 ± 0.01	7.54 ± 0.01***	7.57 ± 0.01***	7.53 ± 0.04***
Controls	7.47 ± 0.01	7.55 ± 0.01***	7.56 ± 0.01***	7.58 ± 0.01***
Perfused vessel density (mm/mm^2^)				
Bowel-O_2_	20.8 [14.9–21.1]	21.2 [17.7–23.1]	21.0 [18.6–22.1]	18.5 [17.1–19.5]
Controls	19.9 [18.2–23.3]	20.0 [18.1–23.8]	18.0 [16.5–22.0]	17.9 [15.7–18.7]

*p < 0.05; **p < 0.01; ***p < 0.001 versus baseline

The administration of O_2_ into the jejunal lumen did not produce any significant difference in any of the respiratory or hemodynamic variables measured over the course of the experiment. No differences were found between bowel O_2_ and control groups at any time points (Fig. 2). Arterial lactate levels decreased during bowel O_2_ administration (*p* = 0.22 versus baseline at 120 min) but rose again over the following 2 h once the oxygen was discontinued (*p* = 0.06 versus baseline at 240 min). Arterial PCO_2_ and pH remained significantly altered throughout the experiment in both groups but did not differ between the groups (Table 2).

Microcirculatory PVD did not change significantly following hypoxemia and no differences were found between the groups at any time points (Table 2).

Cumulative urine output was similar between the two groups at 120 min (3.4 [2.8–6.4] ml/kg in the bowel O_2_ group and 3.1 [2.3–6.0] ml/kg in controls, *p* = 0.32, *n* = 8/group) and 240 min (7.0 [6.1–13.1] ml/kg in the bowel O_2_ group and 6.4 [5.8–12.0] ml/kg in controls, *p* = 0.53, *n* = 5/group).

## Discussion

In our rat model of hypoxemia, the infusion of pure O_2_ gas through a central vein was unfeasible as it induced early death due to pulmonary embolism. The infusion of oxygenated Hartmann’ solution over 3 h was safe; however, it proved ineffective in increasing blood or tissue oxygenation. Similarly, the administration of gaseous O_2_ into the small bowel did not produce any improvement in oxygenation although it was not associated with any unfavorable effects.

Numerous attempts have been made in the past to oxygenate the blood by routes other than the lung. Nysten was probably the first to inject gaseous O_2_ into the veins of a living animal in 1811 [10]. The first administration to a human being dates back to 1902, when Mariani reported an improved pulse and respiration following the infusion of 120 ml O_2_ over 15 min through the dorsalis pedis vein in a patient with tuberculosis, but who died the next day [11]. Several other investigators reported encouraging results in both animals and humans [4, 12–14]; however, these data were not confirmed by others. It became clear that the intravenous administration of pure O_2_ gas could lead to pulmonary embolism and worsened blood oxygenation [15, 16]. It was also suggested that only a small amount of the gas injected could be absorbed as only a minimal surface of gas was in direct contact with the blood due to the coalescence of gas bubbles [16]. Our study in hypoxemic rats supports the lack of safety of intravenous gas infusion. We used an O_2_ infusion rate of 2 ml/kg/h, which would correspond to an infusion rate of 140 ml/h for a human weighing 70 kg. There are case reports showing that the intravenous bolus injection of 100 ml of air can cause cardiac arrest due to fatal pulmonary embolism [17]. Other authors suggest the lethal dose for humans to be 3–5 ml/kg and that a rapid injection of 300–500 ml at a rate of 100 ml/s is fatal [18]. A larger amount of O_2_ may be potentially administered in humans or large animals using peripheral venous accesses and lower rates of infusion without hemodynamic or respiratory sequelae. However, scientific evidences for this are lacking, and the questions as to whether a sufficient volume of gas could be administered safely and absorbed to effectively improve tissue oxygenation remain unanswered.

Despite being safe, the intravenous infusion of oxygenated Hartmann’s solution was not able to induce any increase in blood or tissue PO_2_. In an experimental study in normoxemic rabbits, Kim et al. reported an increase in arterial PO_2_ during the administration of oxygenated Ringer’s lactate through a central vein [19]. However, spontaneous variation cannot be excluded as no control group was used in this study. The PO_2_ of solution at end-experiment in our study was even higher (87.5 ± 1.7 kPa) than that reported by Kim et al. (76.7 kPa); however, it must still be acknowledged that the amount of O_2_ dissolved remains small. In our experiments, at 60 min hypoxemia, the average O_2_ delivery (DO_2_) was 14.4 ± 2.6 ml/min (as calculated from the cardiac output, Hb levels, SaO_2_, and PaO_2_) and the O_2_ consumption (VO_2_) was 5.9 ± 1.2 ml/min (as calculated from the cardiac output and the difference between the arterial and venous O_2_ content). According to the Henry’s law and the ideal gas law, at PO_2_ = 100 kPa and temperature = 20 °C, we can estimate that the amount of O_2_ dissolved in the oxygenated Hartmann’ solution was about 0.03127 ml O_2_/ml. At a fluid infusion rate of 10 ml/kg/h, a rat weighing 300 g received around 0.05 ml/min (3 ml/h) of Hartmann’ solution, containing 0.0016 ml O_2_/min. This represents about the 0.01 % of the total DO_2_ and the 0.027 % of the total VO_2_, thus making an insignificant contribution to systemic oxygenation.

In a guinea pig experiment, Kellogg noted that the dark blood in the portal veins assumed a bright red color soon after the injection of O_2_ into the rectum, suggesting a rapid absorption through the intestinal wall [5]. In a case series, Maruzok et al. reported a significant increase in the PaO_2_/FiO_2_ ratio in patients with acute respiratory distress syndrome following the intestinal insufflation of pure O_2_ gas through a naso-intestinal tube [20]. As the improvement in systemic oxygenation lasted for more than 32 h, direct intestinal absorption of O_2_ seemed unlikely [20]. Again, the lack of a control group prevents definitive conclusions from being drawn. In our study in hypoxemic rats, the administration of pure O_2_ gas into the small bowel was unable to produce any increase in systemic oxygenation, either rapidly (during infusion) or in the 2 h following infusion discontinuation. The liver should have been the first organ to benefit from an intestinal absorption of O_2_ into the portal veins for anatomical reasons. The observed trend towards a reduction in arterial lactate levels during the administration of bowel O_2_ might reflect an improved hepatic metabolism. However, no improvement was seen in liver tPO_2_ during or after the insufflation of O_2_ into the bowel. By administering a 5-ml bolus of pure O_2_ followed by a continuous infusion of 50 ml/kg/h, we infused a total of 27.5 ml of pure O_2_ for a rat weighing 300 g (5 ml + 15 ml/h over 90 min). Even if all this O_2_ had been absorbed in the circulating blood over time, this would have contributed to systemic oxygenation only by about 2 % of the systemic DO_2_ (total DO_2_ over 90 min under FiO_2_ 10 % = 1296 ml) and 4.2 % of systemic VO_2_ (total VO_2_ over 90 min under FiO_2_ 10 % = 531 ml). Our results do not show any evidence supporting the ability of O_2_ to be absorbed in a detectable amount through the intestinal mucosa into the blood.

Other alternatives to pulmonary oxygenation have been explored over the past years. Perfluorocarbon-based O_2_ carriers have been tested using several routes of administration, including intravenous [21], transintestinal [22], and transpleural [23], as well as for liquid ventilation [24]. Nevertheless, none of these agents has been approved for clinical use due to storage, production, and clinical evaluation complications [25]. Kheir et al. manufactured lipid-based O_2_-carrying microbubbles for intravenous infusion that could rescue hypoxemia in asphyxiated rabbits, decreasing the incidence of cardiac arrest and organ injury without inducing any signs of pulmonary embolism [26]. The administration of phospholipid-coated O_2_ microbubbles into the peritoneal cavity was effective in increasing systemic oxygenation in animal models of pneumothorax [27] and asphyxia [28]. The research for simple, non-invasive, and cheap alternatives to pulmonary oxygenation is still ongoing, and further studies are encouraged.

Some limitations of our study need to be acknowledged. Firstly, retrospective theoretical calculations of the amount of O_2_ administered in the different models clearly show that the experimental approach applied was unsuitable to produce any positive results. However, we designed our experiments based on previously published reports [4, 5, 10–16, 19, 20] that represented our proof of concept. Secondly, we constructed an artificial short-term model of hypoxemia by administering a hypoxic gas mixture. Other experimental models of acute respiratory distress syndrome (e.g., by repeated pulmonary lavage [29]) could more closely reproduce the clinical scenario. However, our method allowed a better control of the amount of O_2_ absorbed in the lungs, thus limiting variability in oxygenation parameters. Other potential routes for O_2_ administration were not evaluated, such as the peritoneal route. Mechanical ventilation of the peritoneal cavity was able to improve oxygenation in an experimental model of acute respiratory syndrome in rabbits, inducing both an increase in arterial PO_2_ and a decrease in arterial PCO_2_ [30].

## Conclusions

In hypoxemic rats, the infusion of 2 ml/kg/h of pure O_2_ gas into a central vein induced early death due to pulmonary embolism. However, both the intravenous infusion of oxygenated Hartmann’s solution and the administration of gaseous O_2_ into the small bowel were safe but ineffective in increasing blood or tissue oxygenation. Our study suggests the inadequacy of intravenous oxygenated fluids or intestinal insufflation of O_2_ as alternative ways for O_2_ administration in rats.

## Abbreviations

ABE: Arterial base excess
DO_2_: Oxygen delivery
FiO_2_: Inspired oxygen fraction
HR: Heart rate
MAP: Mean arterial pressure
O_2_: Oxygen
PCO_2_: Carbon dioxide tension
PO_2_: Oxygen tension
PVD: Perfused vessel density
RR: Respiratory rate
SO_2_: Oxygen saturation
SV: Stroke volume
tPO_2_: Tissue oxygen tension
VO_2_: Oxygen consumption
